# Amended diagnosis, mitochondrial genome, and phylogenetic position of *Sphyranura euryceae* (Neodermata, Monogenea, Polystomatidae), a parasite of the Oklahoma salamander[Fn FN1]

**DOI:** 10.1051/parasite/2023025

**Published:** 2023-07-06

**Authors:** Samuel J. Leeming, Christoph Hahn, Stephan Koblmüller, Chris T. McAllister, Maarten P. M. Vanhove, Nikol Kmentová

**Affiliations:** 1 Institute of Biology, University of Graz Universitätsplatz 2 A-8010 Graz Austria; 2 Research Group Zoology: Biodiversity & Toxicology, Centre for Environmental Sciences, Hasselt University Agoralaan Gebouw D 3590 Diepenbeek Belgium; 3 Science and Mathematics Division, Eastern Oklahoma State College 2805 NE Lincoln Road Idabel Oklahoma 74745 United States

**Keywords:** Monogenea, Polystomatidae, *Sphyranura*, Tetrapods

## Abstract

Polystomatidae is a monogenean family whose representatives infect mainly (semi)-aquatic tetrapods. Species of *Sphyranura* Wright, 1879 exhibit ectoparasitism on salamander hosts, with molecular work supporting their inclusion within Polystomatidae, at an early diverging, yet unresolved, position in the clade of otherwise endoparasitic polystomatid parasites of batrachian hosts. Records of representatives of *Sphyranura* are scarce with genetic data only available for *S. oligorchis* Alvey, 1933. Based on detailed morphological examination and comparison with type material, we identified worms belonging to *Sphyranura* infecting Oklahoma salamander (*Eurycea tynerensis*) as *S. euryceae* Hughes & Moore, 1943. Along with an amended diagnosis of *Sphyranura*, we provide the first molecular data for *S. euryceae* in the form of a mitochondrial genome and nuclear (*18S*, *28S* rRNA) markers. Close morphological similarity between the two species of *Sphyranura* is reflected in low genetic divergence. Mitochondrial level comparison reveals instances of tRNA gene rearrangements in polystomatids. Although the phylogenetic reconstruction supports *Sphyranura* as early branching in the lineage of polystomatid monogeneans infecting batrachians, certain nodes remain unresolved.

## Abstract (fr)

Les Polystomatidae sont une famille de monogènes dont les représentants infectent principalement les tétrapodes (semi)-aquatiques. Les espèces de *Sphyranura* Wright, 1879 présentent un ectoparasitisme sur les hôtes salamandres, et des travaux moléculaires soutiennent leur inclusion dans les Polystomatidae, à une position divergente précoce mais non résolue dans le clade des Polystomatidae endoparasites d’hôtes batraciens. Les signalements des représentants de *Sphyranura* sont rares et les données génétiques ne sont disponibles que pour *S. oligorchis* Alvey, 1933. Sur la base d’un examen morphologique détaillé et d’une comparaison avec le matériel type, nous avons identifié les vers appartenant à *Sphyranura* infectant la salamandre de l’Oklahoma (*Eurycea tynerensis*) comme *S. euryceae* Hughes & Moore, 1943. Parallèlement à un diagnostic modifié de *Sphyranura*, nous fournissons les premières données moléculaires pour *S. euryceae* sous la forme d’un génome mitochondrial et de marqueurs nucléaires (ARNr 18S, 28S). La similitude morphologique étroite entre les deux espèces de *Sphyranura* se traduit par une faible divergence génétique. La comparaison au niveau mitochondrial révèle des cas de réarrangements des gènes des ARNt chez les Polystomatidae. Bien que la reconstruction phylogénétique soutienne *Sphyranura* comme un rameau précoce dans la lignée des monogènes Polystomatidae infectant les batraciens, certains nœuds restent non résolus.

## Introduction

Monogenea is a globally distributed class of parasitic flatworms of which the vast majority of species are ectoparasites of actinopterygian and chondrichthyan fishes. However, a number of exceptions to this trend are observed where monogeneans of diverse taxa parasitise sarcopterygian hosts. Examples include *Lagarocotyle salamandrae* Kritsky, Hoberg & Aubry, 1993, of the monotypic family Lagarocotylidae, which infects the Cascade torrent salamander *Rhyacotriton cascadae* Good & Wake [28], *Dactylodiscus latimeris* Kamegai, 1971, a parasite of the coelacanth, representing the monotypic family Neodactylodiscidae [25], three members of Iagotrematidae parasitising two species of salamander [4] and a freshwater turtle [35], and multiple species from the family Gyrodactylidae, including *Gyrodactylus aurorae* Mizelle, Kritsky & McDougal, 1969, *G. catesbeianae* Wootton, Ryan, Demaree & Critchfield, 1993, and *G. jennyae* Paetow, Cone, Huyse, McLaughlin & Marcogliese, 2009 that parasitise amphibian hosts. The subclass Polystomatoinea represents a further such case. Polystomatoineans parasitise exclusively sarcopterygian hosts, with all but a single species parasitising aquatic and semi-aquatic tetrapods. Furthermore, many members of this subclass have also switched from ecto- to endoparasitism in which they typically occupy the urinary bladders of anurans, urodelans and chelonians. Others exhibit ectoparasitism and are found on the conjunctival sacs, pharyngeal cavities, gills, and skin of their host. Polystomatoinea consists of the single family, Polystomatidae [46] with more than 200 species across 31 genera described globally and infecting diverse host taxa [5, 8, 14–18].

The polystomatid genus, *Sphyranura* Wright, 1879 is restricted to North America and its members infect the gills and skin of salamanders. *Sphyranura* consists of *S. osleri* Wright, 1879*, S. oligorchis* Alvey, 1933, *S. polyorchis* Alvey, 1936 and *S. euryceae* Hughes & Moore, 1943. It has been argued, however, that *S. polyorchis* cannot be justified as a separate species from *S. osleri* on the basis of minor morphological differences [41]. *Sphyranura osleri, S. oligorchis* and *S. polyorchis* parasitise the Common mudpuppy (*Necturus maculosus* Rafinesque), with records of *S. oligorchis* also parasitising the Red River waterdog (*Necturus louisianensis* Viosca) [51]. *Sphyranura euryceae* is a parasite of the Oklahoma salamander (*Eurycea tynerensis* Moore & Hughes) [23], a plethodontid salamander endemic to the Ozark Plateau. Adults of this species exhibit alternative life histories with paedomorphic populations associated with chert streambeds where they can access subsurface water year-round and metamorphic populations associated with compact streambeds where such access is not guaranteed [10, 19]. More recently, *S. euryceae* has been observed in the Cave salamander (*Eurycea lucifuga* Rafinesque) [36] and Western Grotto salamander (*Eurycea spelaea* Stejneger) [37]. In general, there is a scarcity of records of representatives of *Sphyranura* and relatively little knowledge about the genus besides morphology and principal host distribution. However, given the intervening decades since Hughes & Moore’s [23] description of *S. euryceae*, advances in staining procedures and microscopy allow for a more detailed morphological examination than was possible at the time of description. Thus, descriptions of representatives of *Sphyranura* often lack some of the morphological information available for more recently studied monogeneans.

*Sphyranura* was long assigned to Sphyranuridae [40], and considered a sister group to Polystomatidae on the basis that its members possess a single pair of haptoral suckers in contrast to three pairs found in other polystomatids [38]. Sinnappah *et al.* [46], however, inferred a phylogeny of Polystomatoinea based on partial sequences of the *18S* rDNA marker, which confidently placed *Sphyranura* within Polystomatidae. These authors further proposed that the morphological differences between *Sphyranura* and Polystomatidae as described above are the result of an evolutionary retention of juvenile characters in adults within *Sphyranura* [46]. However, this phylogeny only included seven representatives of Polystomatidae and a single representative of *Sphyranura.* Furthermore, the position of *Sphyranura* within batrachian polystomes was not well supported. Subsequent work, also based on partial *18S* rDNA sequences, split Polystomatidae into two lineages: one parasitising exclusively amphibians, the other parasitising mainly chelonians. This phylogeny also supported *Sphyranura* as being nested within the lineage of anuran polystomatids, its exact relationships, however, remained unresolved [53]. More recently, Héritier *et al.* [22] inferred the phylogeny of Polystomatidae based on the complete *18S* rDNA sequence, a partial *28S* rDNA sequence and two partial sequences of mitochondrial genes, *cox1* and *12S* rDNA, which supported the division of Polystomatidae into the “Polbatrach” and “Polchelon” (acronyms coined by the authors) lineages with *Concinnocotyla australensis* (Reichenbach-Klinke, 1966), a parasite of the Australian lungfish (*Neoceratodus forsteri* (Krefft)), branching off prior to this split. The former lineage includes all polystomatids of batrachian hosts (Caudata and Anura), whilst the latter includes all polystomatids of chelonian hosts as well as *Nanopolystoma tinsleyi* du Preez, Badets & Verneau, 2014 of the Cayenne caecilian (*Typhlonectes compressicauda* Duméril & Bibron) and *Oculotrema hippopotami* Stunkard, 1924 of the common hippopotamus (*Hippopotamus amphibius* L.). Furthermore, this phylogeny suggested that *Sphyranura* is an early, although unresolved, branching lineage within the “Polbatrach” polystomatids [22]. This phylogeny therefore supported the hypothesis of an origin of Polystomatidae prior to the colonisation of terrestrial environments by tetrapods followed by host-parasite coevolution as different tetrapod lineages diverged [55].

In the present study, we aim to produce an amended diagnosis of *Sphyranura* using various staining techniques to provide morphological characters at a higher resolution than previous work. Further, we provide the first molecular sequences for a member of *Sphyranura* other than *S. oligorchis*, including its mitogenome. Although beyond the scope of the current research, this mitogenome may provide a valuable resource in future phylogenetic studies of Monogenea. Given the unresolved position of *Sphyranura*, questions regarding the number of evolutionary colonisations of caudatan hosts by polystomatid monogeneans remain. We therefore present an updated phylogeny of Polystomatidae, including the new specimens and several other polystomatid taxa made available since the publication of that inferred by Héritier *et al.* [22] in 2015, including those submitted by Du Preez and Verneau [18] in 2020.

## Methods

### Ethics

Specimens were collected under Scientific Collecting Permit (number 021120207) from the Arkansas Game and Fish Commission, Little Rock, Arkansas, USA.

### Sampling

Over three sampling occasions between November 2019 and November 2020, specimens of paedomorphic *E. tynerensis* were collected with an aquatic dipnet at Greathouse Spring in Tontitown, Benton County, Arkansas, USA (Coordinates 36° 8′ 11.1192″ N, −94° 12′ 10.0764″ W). Specimens were placed in habitat water and examined for ectoparasites within 24 h. Salamanders were killed with an overdose of a concentrated solution of tricaine methanesulfonate and their gills and body were examined under a stereomicroscope. When monogeneans were observed on gills, they were removed and relaxed in hot tap water and stored in either 10% neutral-buffered formalin (NBF) or 98% molecular grade ethanol.

### Staining procedure

Seven adult individuals and two larvae used for morphological analysis were selected from those preserved in 10% NBF. These were then stained with various media and mounted on standard microscope slides to be morphologically characterised. The staining procedure included the following steps: Individual worms were first placed in a solution of 70% ethanol to be dehydrated before being overstained using a 1:1 mixture of acetocarmine (or Schneider-acetocarmine in the case of specimens 4, 6 and larva 1) and 70% ethanol (>12 h). The ethanol-acetocarmine mix was then gradually washed out using acid alcohol until internal structures such as testes, ovaries and vesicles were visible under a binocular microscope. At this point, the process was halted by washing in distilled water for 5 min to remove excess acetocarmine. Specimens 1 and 3 were then stained with Astra blue for 40 min before being washed twice in distilled water to wash out residual Astra blue [47]. This step was skipped for specimens 2, 4, 5, 6, 7 and the two larvae. After this, specimens were dehydrated through a series of increasing ethanol concentrations (5 min at 70%, 5 min at 80%, 15 min at 96%, 5 min at 100%) and carboxyl was added. Xylene was then added to clear the specimens and they were mounted on a slide using Canada balsam, ensuring that the specimens were lying flat when the cover slip was added. The slides were then weighted to ensure specimens remained flat and given two weeks on a radiator to dry out. The attachment structures of two individuals were placed on a slide in a drop of water that was subsequently replaced by Hoyer’s medium and covered with a cover slip that was sealed with Glyceel [3].

### Morphological characterisation

**Figure S1.**
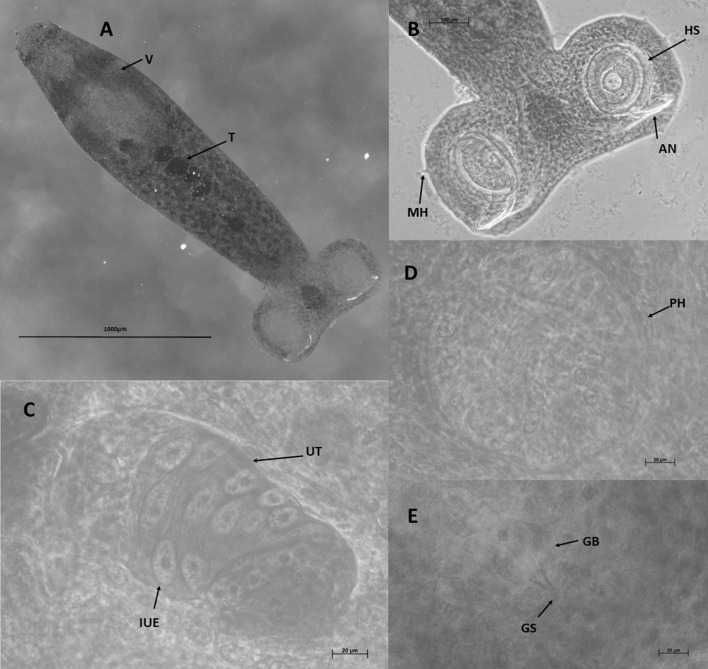
*Sphyranura oligorchis* (AMNH1432.1). A. Full body view, scale bar 1000 μm. B. Haptor, scale bar 100 μm. C. Uterus and intrauterine eggs, scale bar 20 μm. D. Pharynx, scale bar 20 μm. E. Genital bulb and spines, scale bar 20 μm. Abbreviations: PT, point; AN, Anchor; MH, marginal hooklet; V, vesicle; PH, pharynx; GB, genital bulb; GS, genital spines; HS, haptoral sucker; EG, egg; IUE, intrauterine eggs. Figure converted to black and white in Microsoft Publisher.

The morphological part of the study was done using Leica DM 2500 LED microscopes (Leica Microsystems, Wetzlar, Germany) and the software LasX v3.6.0 using Differential Interference Contrast (DIC) and Phase Contrast, where necessary, to gain optimal view of individual anatomical features. In total, 35 morphological characters including hard and soft parts were measured following the terminology of [43]. A comparison of the new specimens with existing type material belonging to *Sphyranura* provided by the American Museum of Natural History (AMNH) was undertaken to further support the species identification of these specimens with re-measurements of type material being undertaken where necessary and possible. The material included two specimens of *S. osleri* (accession numbers AMNH 1427.1 and AMNH 1427.2), one specimen of *S. polyorchis* (accession number AMNH 1431), and three specimens of *S. oligorchis* (accession numbers AMNH 1432.1, AMNH 1432.2 and AMNH 1432.3). Photomicrographs of the type material of *S. oligorchis* (AMNH 1432.1) are provided in Supplementary Figure S1. Parasite voucher material collected as a part of the present study was deposited in the collection of the American Museum of Natural History (AMNH) under accession numbers AMNH_IZC 00382999–AMNH_IZC 00383001 and Hasselt University under accession numbers UH XIX.2.09-XIX.2.15.

### Molecular methods

#### DNA extraction and PCR

Genomic DNA was extracted from four individuals using a Quick-DNA^TM^ Miniprep Plus Kit (Zymo Research Irvine, CA, USA), following the manufacturer’s instructions with minor modifications, specifically: initial incubation overnight, and elution in 2 × 50 μL after 10 min incubation at room temperature, each. DNA was then quantified with a Qubit fluorometer (dsDNA HS assay). The DNA concentration of the individual extracts measured between 0.665 and 1.34 ng/μL. The partial *12S, 28S* and *18S* rRNA genes of four specimens were then amplified and sequenced. Primers used for amplification and sequencing of each gene were selected based on previous work [22, 54] and were as follows: *18S*: IR5/L7, *12S*: 12SpolF1/12SpolR9, for the *28S* two overlapping fragments of unequal length were sequenced. LSU5/IR14 primers were used for larger of these and IF15/LSU3 for the smaller. The reactions were performed in a total volume of 11.2 μL, including 7.05 μL water, 1.0 μL buffer (BioTherm 10× PCR Buffer, 15 mM MgCl_2_), 0.35 μL dNTPs (10 mM), 0.25 μL each of forward and reverse primers (0.1 mM), 0.3 μL *Taq* polymerase (SupraTherm 5 units/μL) and 2.0 μL DNA template. The amplification cycle consisted of a step of 3 min at 95 °C for initial denaturation; 45 cycles of 30 s at 95 °C for denaturation, 30 s at 50 °C for annealing and 1 min at 72 °C for elongation; one final step of 7 min at 72 °C for terminal elongation. The PCR products were visualised on agarose gels in order to verify the success of PCR amplification before sequencing. The PCR products were purified by adding a mixture of 0.5 μL ExoSAP (ExoSAP-IT: Amersham Biosciences) and 1.2 μL water to each and incubating in a thermocycler for 45 min at 37 °C, followed by 15 min at 80 °C. The sequencing reaction was run using a cycle beginning with a single step of initial denaturation for 3 min at 94 °C; 35 cycles of 30 s at 94 °C, 30 s at 50 °C, and 3 min at 60 °C; one final step of 7 min at 60 °C. Sequencing products were purified with SephadexTM G-50 (GE Healthcare Chicago, IL, USA) and sequenced on an ABI 3130xl capillary sequencer (Applied Biosystems, Waltham, MA, USA). All newly generated sequences have been deposited on GenBank (see Table 1).

**Table 1:** List of parasite taxa and their respective host species, country of origin and GenBank accession numbers of the markers used to infer the phylogeny. Taxa marked with * were not included in the phylogeny of Héritier *et al.* [22]. Taxa marked with ** were renamed since the publication of Héritier *et al.* [22] by Fan *et al.* 2020 [20], Du Preez and Verneau 2020 [18], Chaabane *et al.* 2019 [15], Tinsley and Tinsley (2016) [49], Du Preez *et al.* (2022) [17] and Chaabane *et al.* (2022) [14], with original names in brackets. In these cases, the GenBank accession numbers correspond to original names.

				GenBank Accession numbers			
Species	Host species	Country of origin	Infestation site	*12S* rDNA	*18S* rDNA	*28S* rDNA	*Cox1* mtDNA
Polystomatidae							
*Apaloneotrema moleri* *	*Apalone ferox*	USA	Conjunctival sacs	MW029418.1	MW029406.1	MW029412.1	MW029424.1
*Aussietrema spratti* (*Neopolystoma spratti* **)	*Chelodina longicollis*	Australia	Conjunctival sacs	KR856105.1	AJ228788.1	FM992702.1	Z83007.1
*Concinnocotyla australensis*	*Neoceratodus forsteri*	Australia	Gills and Skin		AM157183.1	AM157197.1	
*Diplorchis ranae*	*Glandirana rugosa*	Japan	Urinary bladder	KR856070.1	AM157184.1	AM157198.1	JF699304.1
*Diplorchis shilinensis*	*Babina pleuraden*	China	Urinary bladder	KR856071.1	KR856123.1	KR856141.1	KR856162.1
*Eupolystoma alluaudi*	*Bufo* sp.	Togo	Urinary bladder	KR856072.1	AM051066.1	AM157199.1	FR667558.1
*Eupolystoma vanasi*	*Schismaderma carens*	South Africa	Urinary bladder	KR856073.1	AM157185.1	AM157200.1	FR667559.1
*Fornixtrema elizabethae* *	*Trachemys scripta elegans*	USA	Conjunctival sacs	MW029414.1	MW029402.1	MW029408.1	MW029420.1
*Fornixtrema fentoni* (*Neopolystoma* sp. [R.p.] **)	*Rhinoclemmys pulcherrima*	Costa Rica	Conjunctival sacs	KR856110.1	KR856134.1	KR856153.1	FR822555.1
*Fornixtrema guianensis* * (*Neopolystoma guianensis* **)	*Rhinoclemmys punctularia*	French Guiana	Conjunctival sacs	KY200992.1	KY200987.1	KY200989.1	KY200995.1
*Fornixtrema liewi* (*Neopolystoma liewi* **)	*Cuora amboinensis*	Malaysia	Conjunctival sacs	KR856102.1	KR856128.1	KR856147.1	FR822530.1
*Fornixtrema palpebrae* (*Neopolystoma palpebrae* **)	*Pelodiscus sinensis*	Vietnam	Conjunctival sacs	KR856104.1	FM992696.1	AF382065.1	FR822601.1
*Fornixtrema scorpioides* * (*Neopolystoma scorpioides* **)	*Kinosternon scorpioides*	French Guiana	Conjunctival sacs	KY200993.1		KY200990.1	KY200996.1
*Fornixtrema* sp. [C.s.] (*Neopolystoma* sp. [C.s.] **)	*Chelydra serpentina*	USA	Conjunctival sacs	KR856107.1	KR856131.1	KR856150.1	FR822529.1
*Fornixtrema* sp. [G.p.] (*Neopolystoma* sp. [G.p.] **)	*Graptemys pseudogeographica*	USA	Conjunctival sacs	KR856108.1	KR856132.1	KR856151.1	FR822553.1
*Indopolystoma elongatum* (*Polystoma* sp. [R.a.] **)	*Rhacophorus arboreus*	Japan	Urinary bladder	KR856094.1	AM157190.1	AM157213.1	KR856170.1
*Indopolystoma indicum* (*Polystoma indicum* **)	*Rhacophorus maximus*	India	Urinary bladder	KR856085.1	AM157193.1	AM157216.1	JF699303.1
*Indopolystoma parvum* (*Polystoma* sp. [R.o.] **)	*Rhacophorus omeimontis*	China	Urinary bladder	KR856093.1	AM157189.1	AM157212.1	KR856169.1
*Indopolystoma viridi* (*Polystoma* sp. [R.v.] **)	*Rhacophorus viridis*	Japan	Urinary bladder	KR856095.1	AM157191.1	AM157214.1	KR856171.1
*Kankana manampoka*	*Platypelis pollicaris*	Madagascar	Urinary bladder	KR856074.1	HM854292.1	HM854293.1	JF699307.1
*Madapolystoma cryptica* *	*Guibemantis liber*	Madagascar	Urinary bladder			JN800278.1	JN015518.1
*Madapolystoma ramilijaonae* *	*Guibemantis liber*	Madagascar	Urinary bladder			JN800273.1	JN015525.1
*Madapolystoma* sp. [B.w]	*Blommersia wittei*	Madagascar	Urinary bladder	KR856075.1	FM897290.1	FM897273.1	JF699308.1
*Metapolystoma brygoonis* *	*Ptychadena mascareniensis*	Madagascar	Urinary bladder		FM897287.1	FM897270.1	JN800284.1
*Metapolystoma cachani*	*Ptychadena longirostris*	Nigeria	Urinary bladder	KR856076.1	FM897280.1	FM897262.1	JN800294.1
*Nanopolystoma tinsleyi*	*Typhlonectes compressicauda*	French Guiana	Urinary bladder	KR856077.1	KR856124.1	KR856142.1	KR856164.1
*Neodiplorchis scaphiopi*	*Spea bombifrons*	USA	Urinary bladder	KR856078.1	AM051067.1	AM157201.1	KR856165.1
*Oculotrema hippopotami*	*Hippopotamus amphibius*	South Africa	Conjunctival sacs	KR856120.1	KR856140.1	KR856159.1	KR856178.1
*Parapolystoma bulliense*	*Litoria gracilenta*	Australia	Urinary bladder	KR856079.1	AM157186.1	AM157202.1	KR856166.1
*Pleurodirotrema chelodinae* (*Neopolystoma chelodinae* **)	*Chelodina longicollis*	Australia	Urinary bladder	KR856100.1	KR856126.1	KR856145.1	Z83005.1
*Polystoma australis* *	*Semnodactylus wealii*	South Africa	Urinary bladder		AJ297771.1	AM913872.1	AM913854.1
*Polystoma claudecombesi* *	*Rana angolensis*	South Africa	Urinary bladder		FM897281.1	FM897263.1	
*Polystoma cuvieri*	*Physalaemus cuvieri*	Paraguay	Urinary bladder	KR856080.1	AM051068.1	AM157203.1	AM913862.1
*Polystoma dawiekoki*	*Ptychadena anchietae*	South Africa	Urinary bladder	KR856081.1	AM051069.1	AM157204.1	AM913857.1
*Polystoma floridana*	*Hyla cinerea*	USA	Urinary bladder	KR856083.1	AM157188.1	AM157211.1	AM913870.1
*Polystoma gallieni*	*Hyla meridionalis*	France	Urinary bladder	KR856084.1	AM051070.1	AM157205.1	JF699305.1
*Polystoma integerrimum*	*Rana temporaria*	France	Urinary bladder	KR856086.1	AM051071.1	AM157206.1	JF699306.1
*Polystoma lopezromani*	*Trachycephalus venulosus*	Paraguay	Urinary bladder	KR856087.1	AM051072.1	AM157207.1	AM913863.1
*Polystoma luohetong* (*Polystoma dianxiensis* **)	*Rana chaochiaoensis*	China	Urinary bladder	KR856082.1	KR856125.1	KR856143.1	KR856167.1
*Polystoma marmorati*	*Hyperolius marmoratus*	South Africa	Urinary bladder	KR856088.1	AM051073.1	AM157208.1	AM913858.1
*Polystoma naevius*	*Smilisca baudinii*	Costa Rica	Urinary bladder	KR856089.1	AM157187.1	AM157209.1	AM913864.1
*Polystoma nearcticum*	*Hyla versicolor*	USA	Urinary bladder	KR856090.1	AM051074.1	AM157210.1	AM913865.1
*Polystoma occipitalis* *	*Hemisus marmoratus*	Ivory Coast	Urinary bladder		AM051075.1	FM897264.1	
*Polystoma pelobatis*	*Pelobates cultripes*	France	Urinary bladder	KR856091.1	AM051076.1	KR856144.1	KR856168.1
*Polystoma testimagna*	*Strongylopus fasciatus*	South Africa	Urinary bladder	KR856092.1	AM157194.1	AM157217.1	AM913860.1
*Polystoma umthakathi* *	*Natalobatrachus bonebergi*	South Africa	Urinary bladder			AM913874.1	AM913861.1
*Polystomoidella whartoni* *	*Kinosternon bauri*	USA	Urinary bladder	MW029417.1	MW029405.1	MW029411.1	MW029423.1
*Polystomoides asiaticus*	*Cuora amboinensis*	Malaysia	Pharyngeal cavity	KR856113.1	FM992697.1	FM992703.1	Z83009.1
*Polystomoides australiensis* *	*Emydura krefftii*	Australia	Urinary bladder			Z83012.1	Z83013.1
*Polystomoides cayensis* * (*Neopolystoma cayensis* **)	*Rhinoclemmys punctularia*	French Guiana	Urinary bladder	KY200991.1	KY200986.1	KY200988.1	KY200994.1
*Polystomoides euzeti* (*Neopolystoma euzeti* **)	*Mauremys leprosa*	Algeria	Urinary bladder	KR856101.1	KR856127.1	KR856146.1	KM258887.1
*Polystomoides orbiculare* (*Neopolystoma orbiculare* **)	*Chrysemys picta marginata*	USA	Urinary bladder	KR856103.1	KR856129.1	KR856148.1	FR822531.1
*Polystomoides oris*	*Chrysemys picta marginata*	USA	Pharyngeal cavity	KR856115.1	FM992698.1	FM992705.1	FR822533.1
*Polystomoides soredensis* (*Polystomoides* sp. [T.s.s.] **)	*Trachemys scripta scripta*	USA	Pharyngeal cavity	KR856111.1	KR856135.1	KR856154.1	FR828360.1
*Polystomoides tunisiensis*	*Mauremys leprosa*	Algeria	Pharyngeal cavity	KR856116.1	KR856136.1	KR856155.1	FR822570.1
*Polystomoides* sp. [A.s.] (*Neopolystoma* sp. [A.s.] **)	*Apalone spinifera*	USA	Pharyngeal cavity	KR856106.1	KR856130.1	KR856149.1	FR822527.1
*Polystomoides* sp. [K.l.] (*Neopolystoma* sp. [K.l.] **)	*Kinosternon leucostomum*	Costa Rica	Conjunctival sacs	KR856109.1	KR856133.1	KR856152.1	KR856175.1
*Protopolystoma occidentalis*	*Xenopus muelleri*	Togo	Urinary bladder	KR856121.1	AM051077.1	KR856160.1	KR856179.1
*Protopolystoma xenopodis*	*Xenopus laevis*	South Africa	Urinary bladder	KR856096.1	AM051078.1	AM157218.1	EF380004.1
*Pseudodiplorchis americanus*	*Scaphiopus couchii*	USA	Urinary bladder	KR856097.1	AM051079.1	AM157219.1	KR856173.1
*Pseudopolystoma dendriticum*	*Onychodactylus japonicus*	Japan	Urinary bladder	KR856122.1	FM992700.1	FM992707.1	KR856180.1
*Sphyranura oligorchis*	*Necturus maculosus*	USA	Gills and skin	KR856098.1	FM992701.1	FM992708.1	KR856174.1
*Sundapolystoma chalconotae*	*Hylarana chalconota*	Malaysia	Urinary bladder		AM051080.1	KR856161.1	
*Uropolystomoides bourgati* * (*Polystomoides bourgati* **)	*Pelusios castaneus*	Togo	Urinary bladder		AJ297781.1	AF382068.1	FR822602.1
*Uropolystomoides malayi* (*Polystomoides malayi* **)	*Cuora amboinensis*	Malaysia	Urinary bladder	KR856112.1	AJ228792.1	FM992704.1	Z83011.1
*Uropolystomoides siebenrockiellae* (*Polystomoides siebenrockiella* **)	*Siebenrockiella crassicollis*	Malaysia	Urinary bladder	KR856114.1	FM992699.1	FM992706.1	FR822604.1
*Uropolystomoides* sp. [P.c.] (*Polystomoides* sp. [P.d.] **)	*Pelusios castaneus*	Nigeria	Urinary bladder	KR856119.1	KR856139.1	KR856158.1	KR856177.1
*Uropolystomoides* sp. [P.s.] (*Polystomoides* sp. [P.s.] **)	*Pelomedusa subrufa*	Nigeria	Urinary bladder	KR856118.1	KR856138.1	KR856157.1	KR856176.1
*Uteropolystomoides multifalx* (*Polystomoides* sp. [P.n.] **)	*Pseudemys nelsoni*	USA	Pharyngeal cavity	KR856117.1	KR856137.1	KR856156.1	FR822603.1
*Wetapolystoma almae*	*Rhinella margaritifera*	French Guiana	Urinary bladder	KR856099.1	AM051081.1	AM157220.1	AM913867.1
Outgroup							
*Pseudaxine trachuri*	*Trachurus trachurus*	France	Gills		AM157196.1	AM157222.1	MT666081.1
*Neoheterobothrium hirame*	*Paralichthys olivaceus*	Japan	Buccal cavity	MN984338.1	AB162424.1	LC658937.1	MN984338.1
*Microcotyle* sp.	*Sebastes* sp.	_	Gills	DQ412044.1	AJ287540.1	AF382051.1	DQ412044.1
New specimens							
*Sphyranura euryceae* (SPY1) *	*Eurycea tynerensis*	USA	Gills and skin	OP920607	OP879228	OP879230	OP920607
*Sphyranura euryceae* (SPY2) *				OP920606	OP879229	OP879231	OP920606
*Sphyranura euryceae* (SPY3) *				OP879225		OP879232	
*Sphyranura euryceae* (B2_07) *				OP879226		OP879233	

#### Mitogenome assembly and annotation

DNA extracts of two specimens (SPY1 and SPY2) were sent for whole genome sequencing to commercial sequencing centres. For SPY1, library preparation (Nextera XT, 550 bp insert size) was performed by Macrogen Inc. (Seoul, Korea). For SPY2, library preparation (NEBNext^®^ Ultra IIDNA Library Prep Kit, 550 bp insert size) was done by Novogene (Cambridge, UK). Libraries were sequenced on NovaSeq 6000 systems (2 × 150 bp) at the respective centres. Raw read data were first trimmed using Trimmomatic v.0.38 [9] and the following parameters: a minimum length of 40 bp, a window size of 5 and required quality per window of 15 and a leading and trailing quality of 3. For both specimens, a subsample of 10 000 000 trimmed reads was randomly selected using seqtk v.1.3 [45] with the seed 553353 and fed into the assembly process. A successful assembly of SPY2 was retrieved using GetOrganelle v. 1.7.1 [24]. The first and last 200 bp of this result were joined and trimmed reads were mapped back to this fragment using MITObim v.1.9.1 [21]. Reads mapping full length without any conflict across this tentative junction were taken as verification of circularity. A full-length mitochondrial genome of SPY1 could not be recovered using GetOrganelle, so this sample was assembled *via* MITObim, using the successful SPY2 assembly as a reference. For this result, circularity was confirmed using the script circules.py shipping with MITObim. Annotation was then performed *via* MITOS v.1.0.5 [7] using the genetic code 09 (Echinoderm/Flatworm Mitochondrial). Upon initial visual inspection and comparison of protein-coding genes with those of other monogeneans, it became apparent that there were errors in the start and end positions of many protein coding genes given by MITOS v.1.0.5. The assembly was subsequently submitted to MITOS2 *via* webserver [6]. Start and end positions of protein coding genes as well as start/stop codons were then decided based on visual comparison of the results of MITOS v.1.0.5, MITOS2 and five other monogenean species (*D. hangzhouensis* Zhang & Long, 1987: JQ038227.1, *Neomazocraes dorosomatis* Yamaguti, 1938: JQ038229.1, *Microcotyle caudata* Goto, 1894: MT180126.1, *Polylabroides guangdongensis* Zhang & Yanfg, 2000: JQ038230.1, and *Neoheterobothrium hirame* Ogawa, 1999: MN984338.1) selected based on the highest percentage identity to the mitogenome of SPY2 when performing a BLAST search. This visual inspection further focused on checking for natural open reading frames and stop codon usage. Raw Illumina reads contributing to the mitochondrial genome assemblies were submitted to SRA (accession: SRR22765774–SRR22765775) under BioProject accession PRJNA907756.

In addition to MITOS v.1.0.5, the coordinates and secondary structure of mitochondrial tRNA genes were confirmed using ARWEN v.1.2 [32]. In cases where the coordinates given by MITOS v.1.0.5 did not match those of ARWEN v.1.2, those provided by ARWEN v.1.2 were used, provided a 6–7 bp acceptor stem was present. The *cox1* and *12S* sequences for the samples SPY1 and SPY2 were retrieved from the mitochondrial genomes based on the annotation results from MITOS2. The mitochondrial genome of SPY1 was compared with that of *Diplorchis hangzhouensis* (Accession: JQ038227.1), the only polystomatid species of which the mitochondrial genome is available. Two mitochondrial genomes of *S. euryceae* (SPY1 and SPY2) were deposited on NCBI GenBank under the accession numbers OP920606 and OP920607.

#### Extracting full length 18S and 28S

Whilst only partial *18S* sequences were retrieved *via* Sanger sequencing, the complete *18S* sequence could be extracted from WGS data for the samples SPY1 and SPY2. This was done first using MITObim v.1.9.1 using the *18S* sequence retrieved from Sanger sequencing as an initial seed to extend from the readpool of WGS data, interleaved using BBmap v.38.90 [11]. Barrnap (BAsic Rapid Ribosomal RNA Predictor) v.0.9 [44] was then employed to predict the location of the *18S* sequence within the assembled data. The same method was employed to retrieve the full *28S* sequence, with the partial *28S* sequence, produced *via* Sanger sequencing used as the initial seed. Due to the low coverage of SPY1, an initial assembly could not be retrieved from WGS data using the partial *18S* and *28S* sequences as seeds. Instead, the assembled sequences of SPY2 were used as references for assembly *via* MITObim. Barrnap was subsequently run on the completed SPY1 assemblies to infer the positions of *18S* and *28S*, respectively.

#### Phylogenetic analysis

In addition to sequences obtained from the new specimens, sequences representing a further 66 polystomatid taxa and three non-polystomatid monogeneans were accessed *via* NCBI GenBank. Taxa included in this phylogenetic analysis were selected based on the availability of sequences on NCBI GenBank. A given taxon was included in the analysis on the basis that at least two of the four markers (*12S*, *18S*, *28S* and *cox1*) were present. Partial sequences were included provided they overlap at least in part with the sequences of all other taxa for which sequence data of a given marker was included. In addition to the 55 polystomatid taxa presented in the analysis of Héritier *et al.* [22], sequences from a further 15 polystomatids were included in addition to the new specimens of *Sphyranura*. Species of Gastrocotylidae (*Pseudaxine trachuri* Parona & Perugia, 1890), Diclidophoridae (*Neoheterobothrium hirame* Ogawa, 1999), and Microcotylidae (*Microcotyle* sp.) were selected as an outgroup in line with Héritier *et al.* [22]. Accession numbers of these sequences as well as information on the respective host species, country of origin and site of infection are provided in Table 1.

A maximum likelihood phylogeny was inferred from a subset of the total taxa, representing the clade of polystomatid parasites of batrachian hosts, referred to as the “Polbatrach” clade by Héritier *et al.* [22]. The list of taxa used in this phylogeny is shown in Table 1. Sequences representing these taxa, as well as an outgroup comprising *C. australensis*, were aligned using MAFFT T v.7.464 [26] and trimmed using TrimAl v.1.4.1 [13] in “strict” mode. The four separate alignments were then concatenated into a single alignment using the script concat.py v.0.21 (https://github.com/reslp/concat). PartitionFinder2 [30] selected a GTR+I+G model for the *12S* and *18S* sequences, a TVM+I+G model for the *28S* sequence, and TRN+I+G, TIM+I+G, and GTR+I+G, respectively for the three codon positions of *cox1*. Additionally, phylogenies representing the entire taxa set were inferred *via* two methods. In the first, the four sequence sets were aligned per marker using MAFFT and trimmed using TrimAl “strict mode”. Alignments were inspected visually in AliView v.1.28 [31]. Sequences were concatenated into a single alignment as above. For the second method we performed RNA specific alignment using predicted secondary structure for *18S* and *28S* rRNA markers using R-COFFEE [56], as implemented in T-COFFEE v.11.00 [50]. Since this algorithm does not accept ambiguous nucleotides, we removed any sequences that contained more than one ambiguity characters. For sequences with a single ambiguity character only, the ambiguous character was replaced randomly with one of the candidate characters (custom script replace_IUPAC.py) prior to alignment with R-COFFEE, and the original ambiguity was restored after alignment (custom script restore.py). Alignments were subsequently trimmed as above using TrimAl. The best fitting partitioning schemes for the three ribosomal sequences as well as the three codon positions of the *cox1* gene were selected by PartitionFinder2 using the “greedy search” algorithm. PartitionFinder2 selected a GTR+I+G model for all subsets in the MAFFT alignment, and GTR+I+G for the *12S* and *18S* sequences as well as the three codon positions of *cox1* and the GTR+G model for *28S* in the R-COFFEE alignment. Phylogenetic trees and DNA alignments are openly available in Mendeley Data at https://data.mendeley.com (doi: https://doi.org/10.17632/g286c99yr7.1 & doi: https://doi.org/10.17632/ztjkbv8xf6.1). IQ-TREE v.2.0.7 [39] was then used to infer a Maximum Likelihood phylogeny of all three alignments. Phylogenetic trees were visualised using the web-based tool ITOL (Interactive Tree Of Life) [34].

## Results

### Taxonomic account

Family Polystomatidae Gamble, 1896

Genus *Sphyranura* Poche, 1925

#### Amended diagnosis of *Sphyranura* Poche, 1925

Body elongated with greatest body width found approximately half to two-thirds of distance between haptor and the oral sucker. Body width (measured at widest point) 17–45% of body length with variation between both species and individuals (Table 2). Oral suckers either terminal or subterminal varying in width from 105–300 μm. Single pair of roughly circular haptoral suckers and of anchors, seven pairs of marginal and one pair of acetabular hooks situated at basal end of body. Interior haptoral sucker width accounts for 61–68% of haptor width. Haptor length accounts for 14–19% of body length and haptor width accounts for 26–110% of body width. Vitellaria arranged laterally on both sides of the body extending from region of uterus to peduncle, accounting approximately for two thirds of body length. Testes intercaecal, arranged either in single central row or bunched together along central line of body. Two excretory vesicles at level of genital bulb with dorsal openings. Intestinal bifurcation just posterior to pharynx, fused at level of peduncle. Genital bulb glandular, armed with distally pointed spines. Exhibit ectoparasitism, occupying skin and gills of caudate hosts (*Eurycea tynerensis, E. lucifuga, E. spelaea, Necturus maculosus & N. louisianensis*).

**Table 2:** Morphological measurements in micrometres [μm] of new and previously published specimens of *S. euryceae* [23, 36] including re-measurement of type material of *S. osleri*, *S. oligorchis* and *S. polyorchis* [1]. Range is followed by the mean in parentheses.

Species	*Sphyranura euryceae*				*Sphyranura osleri*		*Sphyranura polyorchis*		*Sphyranura oligorchis*	
Publication	Current work – Adult specimens	Current work – Larval specimens	[23]	[36]	Type material	[1]	Type material	[1]	Type material	[1]
Host	*Eurycea tynerensis*	*Eurycea tynerensis*	*Eurycea tynerensis*	*Eurycea lucifuga* & *Eurycea tynerensis*	*Necturus maculosus*	*Necturus maculosus*	*Necturus maculosus*	*Necturus maculosus*	*Necturus maculosus*	*Necturus maculosus*
No. specimens	7	2	15–30	20	2	_	1	_	3	_
Body length (BL)	1595.45–2554.33 (1946.7)	_	760–2700 (1329)	800–2400 (1620)	893–1562 (1227.5)	2600–4000	2353	2400–3000	1506–2971 (2214)	2500–3500
Greatest body width (BW)	326.14–436.65 (370.844)	_	200–667 (393)	300–600 (420)	254–695 (474.5)	700	741	410–770	496–621 (571.33)	300–400
Oral sucker width (OSW)	203.75–293.65 (245.72)	103.33–127.17 (115.25)	135–320 (196)	155–284 (203)	105.52–229.8 (167.66)	_	266	300	216.1–269 (236.7)	_
Haptor length (HAL)	263.71–366.26 (308.67)	_	141–314 (227)	191–355 (259)	123.16–299 (187.6)	_	372–384 (378)	_	281.91–431.47 (355.06)	_
Haptor width (HAW)	193.53–301.34 (243.99)	_	246–633 (399)	269–767 (463)	101.5–185 (131.3)	_	284–313 (298.5)	_	231.96–392.56 (287.62)	_
Haptoral sucker width (HSW)	78.34–218.5 (151.2)	61.83–65.59 (63.71)	_	_	81.53–103 (91.3)	_	180–186 (183)	_	119.87–391.95 (215.04)	_
Inter-haptoral distance (IHD)	101.55–150.25 (122.21)	_	_	_	54.86	_	155.7	_	89.64–324.67 (223.7)	_
Marginal hooklet length (MHL)	13.77–29.43 (23.25)	_	_	_	_	_	_	_	22.48–37.4 (29.94)	25
Anchor length (AL)	110.15–182.15 (138.67)	_	_	_	102.42–194.58 (162.85)	200	186.3–196.9 (191.6)	_	158.75–219.5 (182.39)	260
Length to notch (LN)	79.8–80.3 (80.05)	_	_	_		_	_	_	_	_
Outer root length (ORL)	81.6–82.44 (82.02)	_	_	_		_	_	_	_	_
Inner root length (IRL)	67.49–70.02 (68.76)	_	_	_		_	_	_	_	_
Point length (PL)	42.64–50.6 (46.62)	_	_	_		_	_	_	_	_
Pharynx length (PHL)	109.8–177.71 (145.67)	48.73–91.57 (70.15)	53–153 (93)	_	81.44	_	98	_	120.95–168.2 (147.92)	_
Pharynx width (PHW)	96.26–175.7 (124.94)	53.73–78.75 (66.24)	60–153 (117)	_	73.71	_	146	150	104.51–158.21 (137.4)	_
Vesicle length (VL)	62.39–138.56 (96.94)	30.32	_	_	_	_	_	_	59.1–74.7 (66.9)	_
Vesicle width (VW)	22.58–254 (76.3)	22.01	_	_	_	_	_	_	30.8–32.1 (31.45)	_
Testes length (TL)	57.65–93.58 (76.87)	70.31	37–98	53–98 (77)	27.1–70.4 (48.75)	_	98	_	59.6–94.5 (82.07)	100
Testes width (TW)	46.46–74.4 (58.47)	81.95	30–105	78–120 (102)	17.6–97.3 (57.45)	_	113	_	74.8–128.1 (92.97)	80
Testes number (TN)	5–7 (6)	6	_	_	10–14 (12)	_	20	_	5–6 (5.67)	
Ovary length (OVL)	98.13–171.84 (125.51)	56.05	_	_	_	100	89	85	46.89–177.6 (113)	65–75
Ovary width (OVW)	73.04–103.58 (90.16)	68.9	_	_	_	160	51	65	70.68–111.78 (91.59)	100
Egg length (EL)	257.45–291.49 (274.47)	_	240–373 (308)	254–282 (268)	321	364	316	_	356.98	280–410
Egg width (EW)	144.62–160.02 (152.32)	_	180–240 (199)	145–217 (190)	151	247	193	_	162.28	220–260
Intrauterine eggs (IUE)	Yes/No/No/No/Yes	Yes	Yes	Yes	No	Yes	No	_	Yes/No/No	Yes
Genital bulb width (GBW)	23.65–48.56 (28.99)	21.71	30–61 (46)	_	_	_	_	_	51.12–54.31 (52.55)	_
Genital spines number (GSN)	8	6	7–9 (8)	_	_	_	_	_	8	_
Genital spines length (GSL)	15.18–24.74 (17.64)	25.41	22–29 (25)	_	_	_	_	_	13.65–21.34 (18)	_
HAL/BL (%)	13.98–19.49 (16.18)	_	17	16	13.79–19.14 (16.47)	_	16.32	_	14.52–18.72 (16.68)	_
HAW/BW (%)	59.68–70.40 (64.9)	_	101.5	110.2	26.6–41.14 (33.87)	_	40.28	_	42.85–57.36 (50.84)	_
PHL/BL (%)	6.31–11.14 (7.72)	_	7	_	9.12	_	4.16	_	5.66–8.03 (6.94)	_
TL/BL (%)	3.1–5.87 (4.19)	_	_	4.8	3.03–4.5 (3.765)	_	4.16	_	3.1–4.36 (3.8)	_
OVL/BL (%)	4.43–8.27 (6.79)	_	_	_	_	_	3.78	_	3.11–8.2 (5.06)	_
BW/BL (%)	17.1–26.95 (19.67)	_	29.6	25.9	28.44–44.49 (36.47)	_	31.49	_	20.09–41.24 (28.08)	_
HSW/HAW (%)	46.6–75.57 (61.55)	_	_	_	55.68–81.28 (68.48)	_	60.66	_	50.32–89.87 (68.16)	_

#### *Sphyranura euryceae* Hughes & Moore, 1943

*Type-host*: *Eurycea tynerensis* Moore & Hughes, 1939

*Other hosts*: *Eurycea lucifuga* Rafinesque, 1822, *Eurycea spelaea* Stejneger, 1892

*Type-locality*: Pea Vine Creek, Cherokee County, Oklahoma, USA

*Other locality*: Greathouse Spring in Tontitown, Benton County, Arkansas, USA

*Type-specimens: Holotype*: US National Parasite Collection no. 36873 Hughes & Moore [23]. *Syntype*: USNM 1337573 Hughes & Moore [23]. *Vouchers*: USNM 1376383, McAllister [36], USNM 1398045 and 1398048 Bursey, AMNH AMNH_IZC 00382999-AMNH_IZC 00383001 present study, UH XIX.2.09-XIX.2.15 present study.

*Infection site*: Skin mainly at the base of legs, and external gills.

*Infection parameters*: Current study – in 2019, 12 specimens of *E. tynerensis* out of 27 infected (prevalence = 44.4%) with one or two individuals per host; in 2020, two out of six specimens of *E. tynerensis* infected (prevalence = 33.3%) with one individual. McAllister [36] reported infection in ten out of ten specimens of *E. lucifuga*, and ten out of ten specimens of *E. tynerensis* (prevalence = 100%). McAllister [37] reported infection in 37 of 74 specimens of *E. tynerensis* and one of two specimens of *E. spelaea* (prevalence = 50%).

*Representative DNA sequences*: GenBank accession numbers OP879228-OP879229 (*18S* rDNA), OP879230-OP879233 (*28S* rDNA), OP879225-OP879226 (*12S* rDNA), OP920606-OP920607 (mitochondrial genome).

##### Morphological characters

Small fusiform worms with a subterminal oral sucker at one end of the body and a single pair of haptors at the other. The oral sucker is followed by the pharynx which is wide and oval tapering to a narrow point at the anterior end. With the exception of the haptors, the body’s widest point is situated roughly two thirds of the way along the body starting from the peduncle. From the peduncle to this widest point of the body is situated a mass of vitellaria. Testes were observed in four of the seven adult specimens, numbering between 5–7 per individual and were arranged in a single line along the centre of the body and were in some cases at least partially obscured by the vitellaria. The ovary was observed in all adult specimens in the study, situated anterior to the testes and vitellaria. Intra-uterine eggs were observed in two specimens. A spherical genital bulb with conical spines is situated anterior to the ovary and connected to the testes *via* the vas deferens, although this latter was only observable in one specimen. Two roughly circular haptoral suckers were situated laterally to the posterior end of the body. Each haptor possessed several marginal hooklets in addition to a much larger anchor which exhibits an accessory sclerite at the base of a larger recurved hook and a deep, triangular cut between the inner and outer roots. Measurements of the aforementioned features, both on new specimens and type material, as well as previous data on *Sphyranura* spp. are presented in Table 2. In addition to the seven adult specimens, morphological characteristics of two larvae were taken. Micrographs showing morphological features of *S. euryceae* are presented in Figure 2.

**Figure 1:**
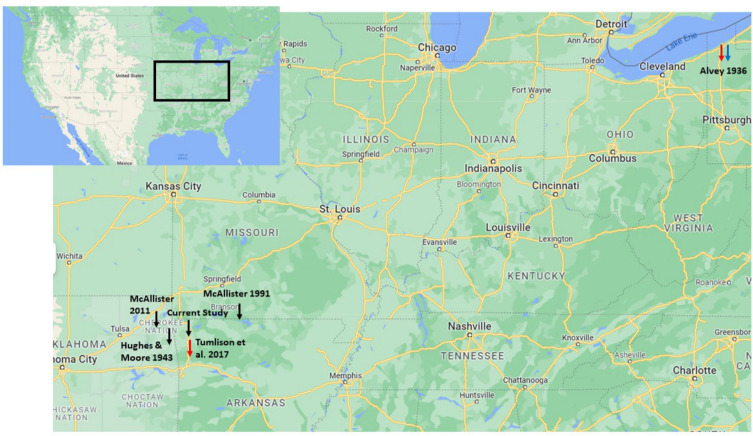
Geographic distribution of published records of *Sphyranura* where sampling location is available. Records of *S. euryceae, S. oligorchis* and *S. polyorchis* are marked in black, red, and blue, respectively.

**Figure 2:**
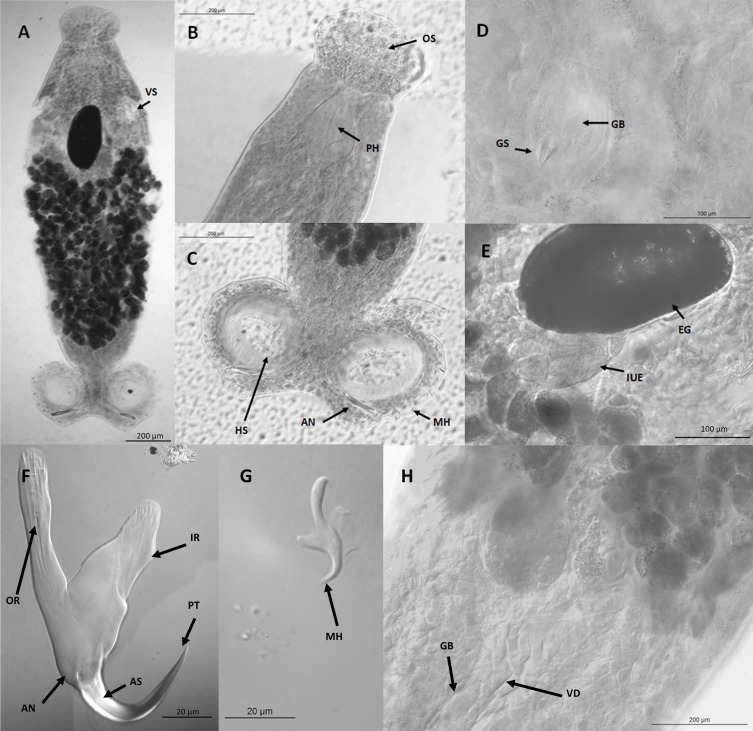
Microphotographs of *Sphyranura euryceae*. A. Full body view, scale bar 200 μm. B. Oral sucker and pharynx, scale bar 200 μm. C. Haptor, scale bar 200 μm. D. Genital bulb and spines, scale bar 100 μm. E. Egg, scale bar 100 μm. F. Anchor, scale bar 20 μm. G. Marginal hooklet, scale bar 20 μm. H. Vas deferens, scale bar 200 μm. Abbreviations: PT, point; AN, Anchor; AS, accessory sclerite; IR, inner root; OR, outer root; MH, marginal hooklet; VS, vesicle; PH, pharynx; OS, oral sucker; GB, genital bulb; GS, genital spines; HS, haptoral sucker; EG, egg; IUE, intrauterine eggs; VD, vas deferens. Figure converted to black and white in Microsoft Publisher.

##### Differential diagnosis

*Sphyranura euryceae* may be distinguished from congeners on a number of morphological features. First, the overall body shape is more elongated than that of congeners (body width as a proportion of body length = 20% vs *S. osleri* = 36%, *S. polyorchis* = 31% and *S. oligorchis* = 28%), although there is some degree of overlap with *S. oligorchis*, but not with *S. osleri* and *S. polyorchis*. Further, haptor width as a proportion of body width is much greater in *S. euryceae* compared to the others (*S. euryceae* = 65% vs *S. osleri* = 34%, *S. polyorchis* = 40% and *S. oligorchis* = 51%). The oral sucker of *S. euryceae* is sub-terminal rather than terminal as in the other members of the genus. The mean anchor length of *S. euryceae* is also less than that of congeners although there is overlap between all species in this trait.

### Mitochondrial genome

**Figure 3:**
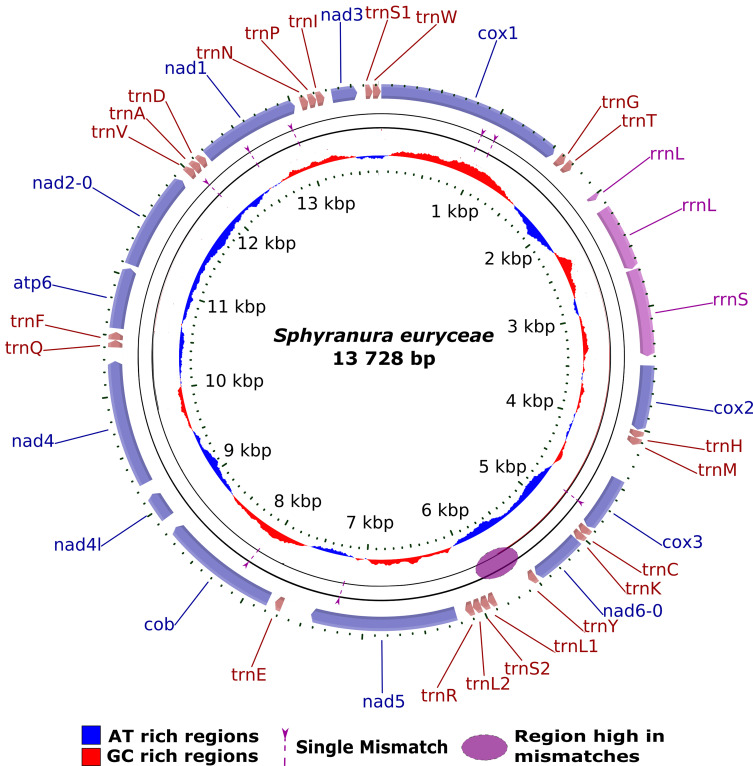
Visualisation of the annotated mitochondrial genome of *S. euryceae*. The mitogenome (13 728 bp) contains 12 protein-coding genes, two ribosomal RNA genes, and 22 tRNA genes. Protein-coding genes are labelled in purple, ribosomal RNA genes in pink, and tRNA genes in brown. Mismatches between the samples SPY1 and SPY2 are indicated by dashed arrows and the region high in mismatches is indicated by the purple oval. AT rich regions are shown in blue in the inner circle whilst GC rich regions are shown in red.

Mitochondrial genomes were assembled for the samples SPY1 and SPY2, a representation of which is presented in Figure 3. The assembly of SPY2 was performed using GetOrganelle from a subsample of 10 million reads, 41 406 of which were used post-filtering to assemble the mitochondrial genome. The assembly had a total length of 13 728 bp and an average coverage of 201. Annotation of this assembly reveals the presence of 12 protein coding genes (the absence of *atp8* is a characteristic of Neodermata [48]). Three non-coding regions with elevated AT content were found between *cox1* and *rrnL* (469 bp, 78% AT), *nad6* and *nad5* genes (738 bp, 79% AT) and *cox2* and *cox3* genes (439 bp, 74% AT). *De novo* assembly of SPY1 was attempted using both GetOrganelle and MITObim but did not successfully produce a full-length mitochondrial genome. However, when assembled using MITObim using the assembly of SPY2 as a reference, a full mitochondrial genome was recovered from a subsample of 10 million reads, 12 310 of which were mitochondrial. The two sequences were nearly identical with the following exceptions shown in Table 3. In addition to these differences, there was a region of high dissimilarity between the positions 5545 and 5996. This dissimilarity was likely due to the presence of AT repeats which rendered this region difficult to assemble. Coverage differed between the two samples and is indicated in Table 4. A comparison of this mitochondrial genome with that of *D. hangzhouensis* is provided in Table 5. Overall, the two tRNA-genes missing in the original annotation of *D. hangzhouensis*, *trnV* and *trnA*, were found (see Table 5). Gene order differences of adjacent features between the two polystomatid species include the following two cases. In *S. euryceae*, *trnS2* precedes *trnL2* whereas in *D. hangzhouensis*, this is reversed. In *S. euryceae*, we see the sequence *trnK*/*nad6/trnY* whereas in *D. hangzhouensis*, we see *trnY/nad6/trnK*.

**Table 3:** Positions of mismatches between the sequences of SPY1 and SPY2 and the gene in which these are found.

Position(s)	946	1021	4812–4814	7235	8115	12 040	12 513	12 918
SPY1	A	A	TAA	T	G	G	C	G
SPY2	T	T	–	C	A	A	T	T
Gene	*cox1*	*cox1*	*cox3*	*nad5*	*cob*	*trnA*	*nad1*	*nad1*

**Table 4:** Library preparation kits and mitochondrial coverage of the sequences of SPY1 and SPY2.

ID	SPY1	SPY2
Library prep kit	Nextera XT	NEBNext^®^ Ultra IIDNA
Subsample	10 million	10 million
Mitochondrial reads	12 310	41 406
Average coverage	59.91	386.04

**Table 5:** Comparison of mitochondrial genomes of *Sphyranura euryceae* (SPY2 – NCBI GenBank accession number OP920606) and *Diplorchis hangzhouensis* (NCBI GenBank accession number JQ038227.1) including start and end positions of each feature, the start and stop codons of protein-coding genes and anticodons of tRNA genes. Positions given for *D. hangzhouensis* are as provided on NCBI. However, the trnA and trnV genes were not included on the NCBI annotation but were found in the present study, when reannotating the *D. hangzhouensis* genome with MITOS2 (indicated with *) or Arwen (**).

*Sphyranura euryceae*				*Diplorchis hangzhouensis*			
Feature	Position	Start/Stop Codon	Anticodon	Feature	Position	Start/Stop Codon	Anticodon
*cox1*	2–1572	ATG/TA–		*cox3*	1–771	ATG/TAG	
*trnG*	1583–1650		TCC	*trnC*	772–837		GCA
*trnT*	1662–1727		TGT	*trnY*	857–922		GTA
*rrnL*	2146–2689			*trnK*	939–1006		CTT
*rrnS*	2699–3421			*nad6*	1051–1458	ATG/TAG	
*cox2*	3422–4037	ATG/T–		*trnL1*	1466–1534		TAG
*trnH*	4038–4099		GTG	*trnL2*	1849–1920		TAA
*trnM*	4099–4161		CAT	*trnS2*	1926–1997		TGA**
*cox3*	4180–4941	ATG/TAG		*trnR*	2014–2087		TCG
*trnC*	4940–5003		GCA	*nad5*	2095–3615	ATT/TTT	
*trnK*	5004–5067		CTT	*trnE*	3606–3671		TTC
*nad6*	5065–5520	ATG/TAA		*cob*	3679–4815	ATG/TAA	
*trnY*	5522–5582		GTA	*nad4l*	4816–5083	ATT/TAA	
*trnL1*	5903–5963		TAG	*nad4*	5047–6306	ATG/TAA	
*trnS2*	5965–6029		TGA	*trnQ*	6309–6372		TTG
*trnL2*	6031–6096		TAA	*trnF*	6377–6440		GAA
*trnR*	6097–6163		TCG	*atp6*	6422–7006	ATG/TAG	
*nad5*	6245–7694	ATG/T–		*nad2*	7157–8068	GTG/TAT	
*trnE*	7696–7759		TTC	trnV *	7896–7967		TAC **
*cob*	7764–8903	ATG/TAA		*trnA* *	7965–8033		TGC
*nad4l*	8896–9150	ATG/TAG		*trnD*	8043–8111		GTC
*nad4*	9111–10 382	GTG/TAA		*nad1*	8115–9023	ATG/TAG	
*trnQ*	10 380–10 442		TTG	*trnN*	9023–9087		
*trnF*	10 507–10 444		GAA	*trnP*	9091–9160		GTT
*atp6*	10 508–11 074	ATG/TAA		*trnI*	9163–9234		TGG
*nad2*	11 074–11 942	ATG/TA–		*nad3*	9235–9558	ATG/TAG	GAT
*trnV*	11 954–12 020		TAC	*trnS1*	9557–9617		
*trnA*	12 018–12 086		TGC	*trnW*	9626–9690		GTC **
*trnD*	12 088–12 152		GTC	*cox1*	9695–11 279	ATG/TA(A)	TCA
*nad1*	12 152–13 061	GTG/TAA		*trnG*	11 280–11 347		
*trnN*	13 058–13 121		GTT	*trnT*	11 348–11 414		TCC
*trnP*	13 126–13 190		TGG	*rrnL*	11 415–12 398		TGT
*trnI*	13 192–13 260		GAT	*rrnS*	12 399–13 136		
*nad3*	13 262–13 570	GTG/TAG		*cox2*	13 137–13 751	ATG/TAA	
*trnS1*	13 600–13 658		GCT	*trnH*	13 754–13 824		GTG
*trnW*	13 661–13 725		TCA	*trnM*	13 834–13 898		CAT

### Phylogeny

**Figure 4:**
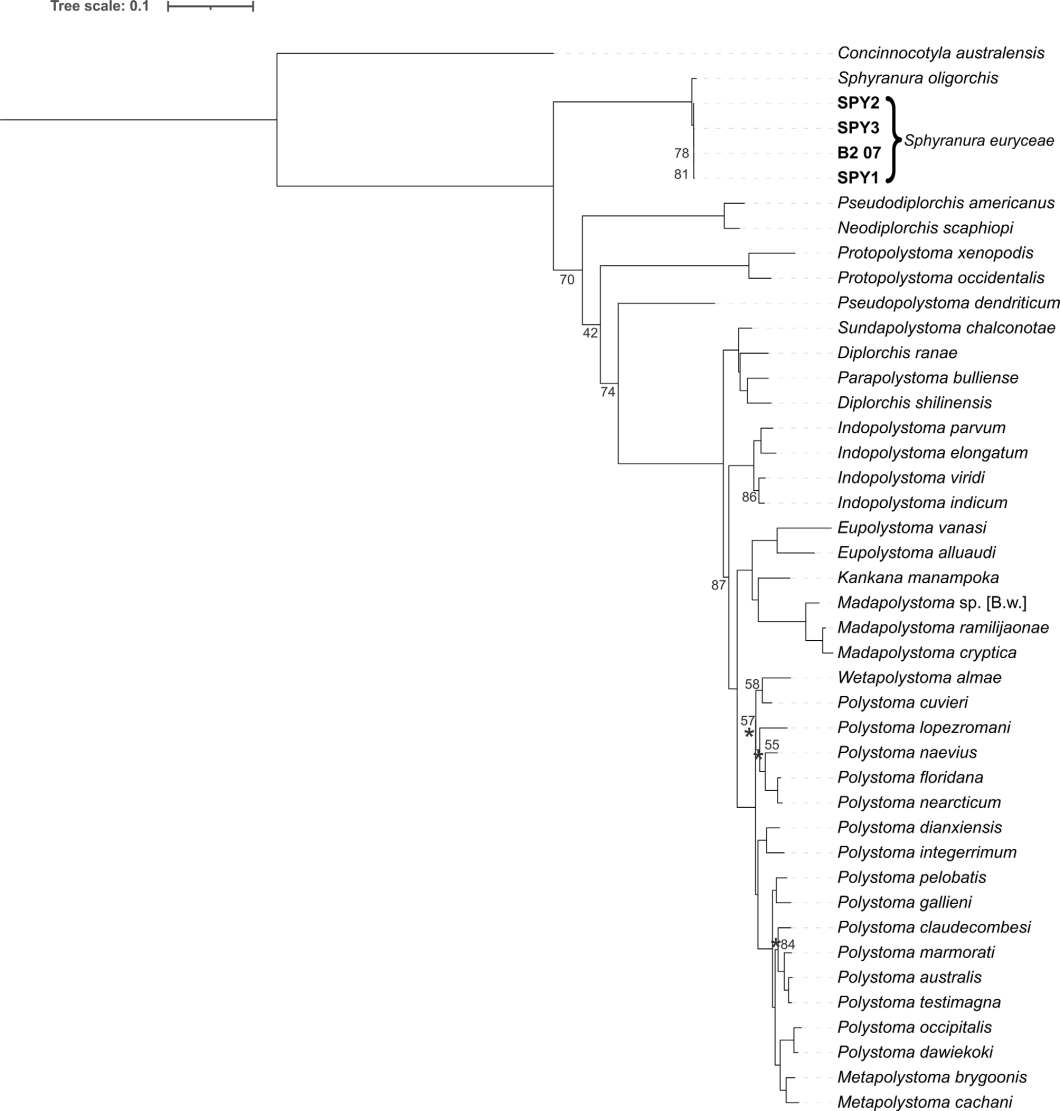
Maximum Likelihood tree of the ‘Polbatrach’ lineage of Polystomatidae based on four concatenated nuclear (*18S* and *28S* rRNA) and mitochondrial (*12S* rRNA and *cox1*) gene portions aligned using MAFFT. Bootstrap values are indicated at the nodes where support is less than 90. Where it is unclear to which node a bootstrap value belongs, this is indicated with an asterisk.

**Figure 5:**
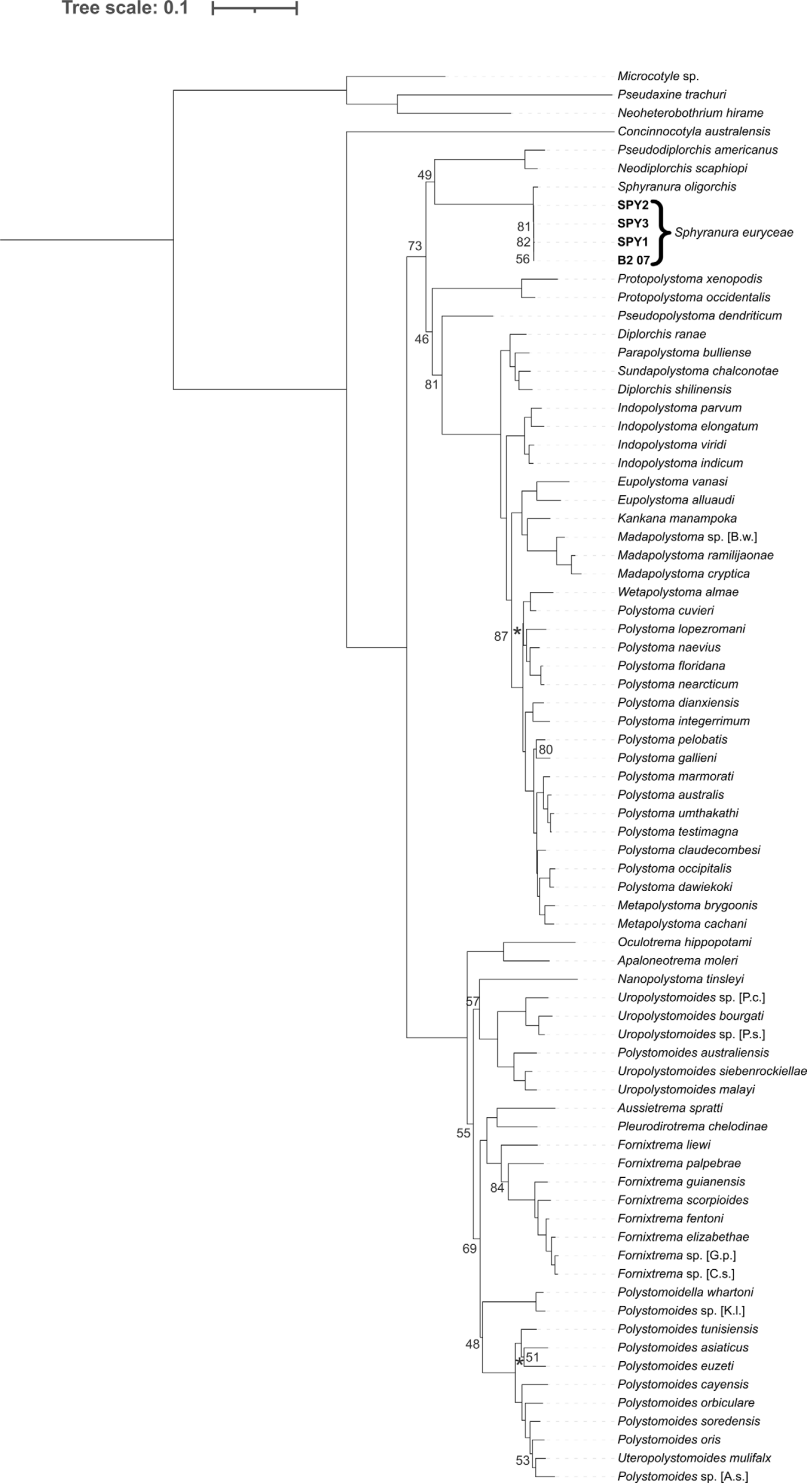
Maximum Likelihood tree of Polystomatidae based on four concatenated nuclear (*18S* and *28S* rRNA) and mitochondrial (*12S* rRNA and *cox1*) gene portions aligned using MAFFT. Bootstrap values are indicated at the nodes where support is less than 90. Where it is unclear to which node a bootstrap value belongs, this is indicated with an asterisk.

**Figure 6:**
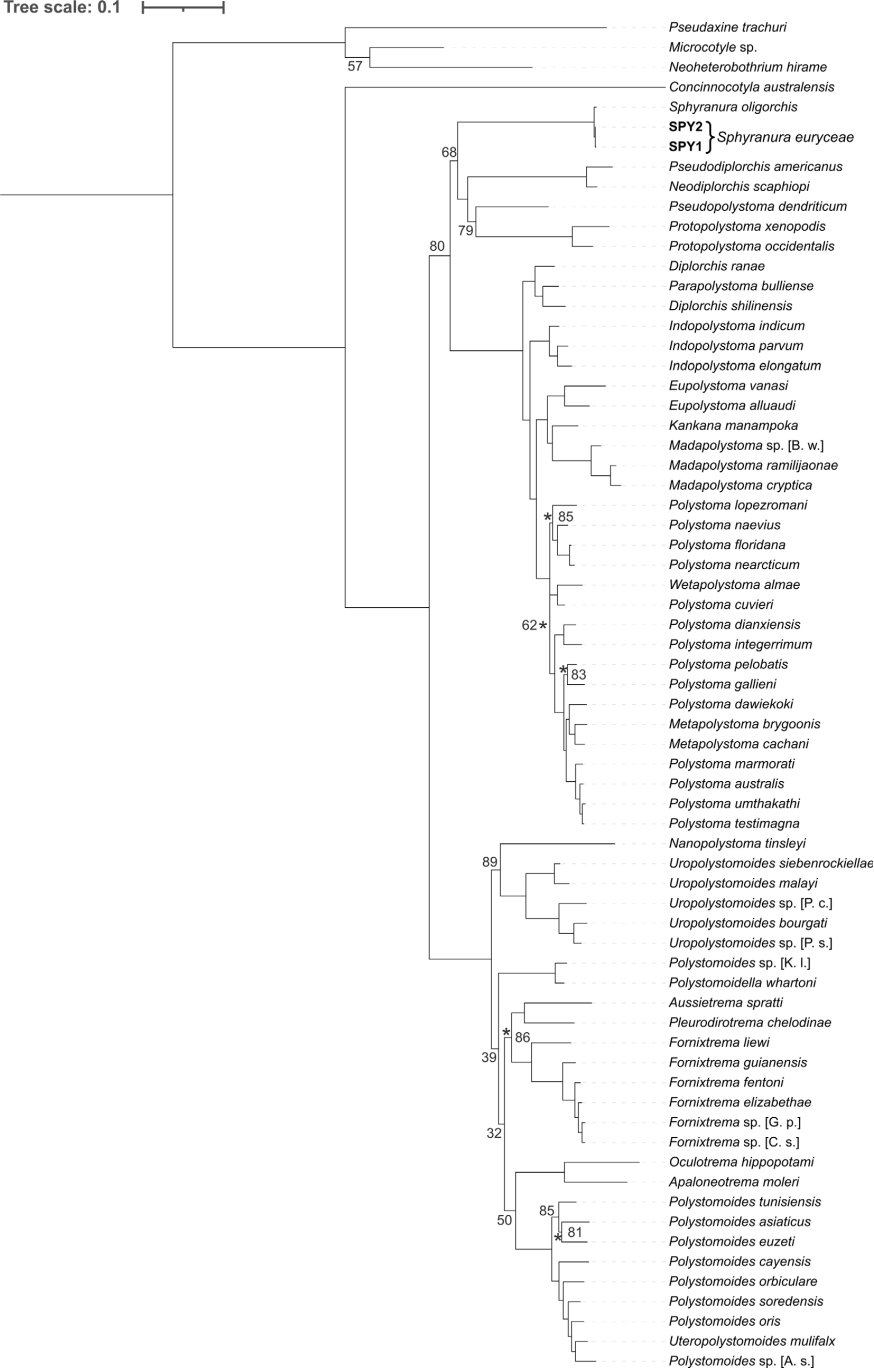
Maximum Likelihood tree of Polystomatidae based on four concatenated nuclear (*18S* and *28S* rRNA) and mitochondrial (*12S* rRNA and *cox1*) gene portions aligned using R-COFFEE. Bootstrap values are indicated at the nodes where support is less than 90. Where it is unclear to which node a bootstrap value belongs, this is indicated with an asterisk.

Sequences of *S. euryceae* were highly similar to those of *S. oligorchis* with percentage identities of 93.6% for *12S* (481 bp), 99.4–99.5% for *18S* (2009 bp), 100% for *28S* (1411 bp) and 96.9–97.4% for *cox1* (395 bp). Intraspecific variation within *S. euryceae* reaches 0.002% in the portion of *cox1* region. A Maximum Likelihood tree was inferred from a restricted taxa set representing the 42 polystomatids that make up the ‘Polbatrach’ clade and were aligned using MAFFT (Figure 4). A further two Maximum Likelihood trees were inferred from 77 taxa (including 74 polystomatids and three non-polystomatid monogeneans) based on alignments produced in MAFFT (Figure 5) and R-COFFEE (Figure 6) and in all trees, specimens of *S. euryceae* formed a monophyletic group that formed a sister-group relationship with *S. oligorchis* at an early branching, but unresolved position within the clade dubbed ‘Polbatrach’ by Héritier *et al.* [22]. However, the three trees present conflicting topologies and are characterised by low support values, making it impossible to determine the true evolutionary relationship of *Sphyranura* to other polystomatid parasites of batrachian hosts.

## Discussion

### Status of *Sphyranura euryceae*

We provide an amended diagnosis of *Sphyranura* and obtained the first-ever molecular sequence data for *S. euryceae*. The divergence between *S. euryceae* and *S. oligorchis* appears rather low compared to other congener polystomatid species. Species of *Metapolystoma* for instance exhibited 7.1–14.9% divergence in *cox1* [29]. However, given that the two species are found on different hosts with non-overlapping ranges as well as the observed morphological differences, we argue that these represent two species, as traditionally described. The high molecular similarity of these sequences indicates that the split between these species was indeed recent. Comparison at the mitochondrial genome level revealed instances of gene order differences in polystomatids. *Sphyranura* was long thought to belong to Sphyranuridae. This was contradicted by the first molecular phylogenies, which placed it at an early-diverging, yet currently unresolved, position in the clade of polystomatids infecting batrachian hosts [22, 53]. The inclusion of a second species of *Sphyranura* as well as 15 polystomatid taxa not included in the phylogeny by Héritier *et al.* [22] indicates an early branching *Sphyranura* within this clade. However, as in previous phylogenies [22, 53] support for this position was ambiguous.

### Morphological comparison of *Sphyranura* spp.

Morphological analysis of the new specimens of *S. euryceae* and comparison of these with type material of *S. osleri*, *S. oligorchis* and *S. polyorchis* revealed high levels of both variability between conspecific individuals and overlap between each of the four species. It is important to note that individuals measured in this study as well as previous studies may well represent different life stages and may well have experienced different conditions prior to collection. Furthermore, the body tissues of monogeneans, with the exception of the sclerotised attachment organs, are soft and may not lie completely flat during slide preparation. For these reasons, relative measurements should be used rather than absolute measurements for species differentiation. That said, the following features provided an informative diagnosis of *S. euryceae*: an overall body shape which was elongated compared to congeners; greater haptoral sucker width in relation to body width; and a sub-terminal, rather than terminal oral sucker. Finally, anchor length of *S. euryceae* was also less than that of congeners. It should also be noted that type material measured in this study represented only a single individual of *S. polyorchis*, of which many features were impossible to observe and measure. *Sphyranura osleri* was represented by two individuals, both deposited in 1879 and perhaps due to their age, many features were again impossible to measure. Based on this, no definite conclusion should be drawn regarding the validity of *S. polyorchis* as questioned by Price [42].

### Mitochondrial genome of *Sphyranura euryceae*

We provide the first available mitochondrial genome for *Sphyranura* and the second only for Polystomatidae. This mitochondrial genome may provide particular value for future phylogenetic work due to the fact that currently available mitogenomes for the sub-class Polyopisthocotylea are all, with the exception of *D. hangzhouensis*, from the order Mazocraeidea [2]. Furthermore, a second polystomatid mitogenome allows for the first insights on the gene order rearrangements in Polystomatidae. As with the majority of flatworm mitochondrial genomes available so far, 12 protein coding genes were found, with *atp8* being absent [48]. A further 22 tRNA genes and the genes coding for both the large and small subunits of the mitochondrial rRNA were present. Comparison with the mitochondrial genome of *D. hangzhouensis* reveals similar gene order, with two instances of rearrangement in the order of adjacent tRNA genes between the two species. However, the order of protein coding genes was conserved between the two species. This is consistent with observations in other monogenean families such as Dactylogyridae [12, 27] and Capsalidae [57], which exhibit rearrangements in the order of tRNA genes between species but generally not in protein coding genes. However, this should not be taken at face value as gene order in some groups of flatworms has been shown in some instances to be highly variable. Rearrangements in protein coding gene order have, for example, been observed within the genus *Schistosoma* [33]. Whether, and to what extent, such rearrangements exist in Polystomatidae therefore warrants further study as additional mitochondrial genomes become available. We identify differences in start/stop codon usage in eight of 12 protein coding genes between the two polystomatids. Furthermore, the abbreviated stop codon (TA-) was used in *cox1* of *S. euryceae*, whereas this stop codon was TAA in *D. hangzhouensis*. The fact that the mitochondrial genome of SPY1 could not be assembled *de novo* indicates that when performing library preparation with low input data, the NEBNext^®^ Ultra IIDNA Library Prep Kit is preferable to Nextera XT.

#### Phylogenetic position of *Sphyranura*

As first suggested by Sinnappah *et al.* [46] and supported by Héritier *et al.* [22], our phylogeny places *Sphyranura* within the ‘Polbatrach’ clade of Polystomatidae, rendering Sphyranuridae invalid. Although not fully supported, our phylogeny indicates *Sphyranura* to be an early branching lineage of the ‘Polbatrach’ clade. Moreover, two independent transitions to caudatan hosts are suggested, though low support of the early branching lineages restricts us from drawing final conclusions. *Sphyranura oligorchis* and *S. euryceae* formed a monophyletic group with little distance between them. Given this phylogenetic proximity and the overlap of many morphological characters seen here, it seems likely that the divergence of the two species occurred in the relatively recent past, following the acquisition of alternative host species by the ancestor of *S. euryceae*.

Apart from members of *Sphyranura*, *Pseudopolystoma dendriticum* Osaki, 1948, also parasitises the Japanese clawed salamander, *Onychodactylus japonicus* (Houttuyn). The two species are not closely related, thus indicating two independent acquisitions of urodelan hosts. Unlike the hosts of *Sphyranura, O. japonicus* goes through a full metamorphosis, during which larvae lose their external gills [52]. As a result, the acquisition of caudatan hosts by the ancestor of *P. dendriticum* was accompanied neither by a shift to ectoparasitism nor a retention of larval morphology as seen in *Sphyranura*.

As in the results of Héritier *et al.* [22], the interrelationships between *Sphyranura*, *Protopolystoma*, (*Neodiplorchis* and *Pseudodiplorchis*) and *Pseudopolystoma* are poorly resolved, which leaves the topology of Polystomatidae ambiguous. Given the variation in topologies we observed when using different alignment algorithms and trimming parameters, it is unlikely that future efforts to increase taxon sampling breadth will resolve the phylogeny of this group with the markers used in this study. A more important step in resolving this phylogeny is access to more data, preferably on the genomic scale, which to date is lacking.

## Supplementary material

The Supplementary materials of this article are available at https://www.parasite-journal.org/10.1051/parasite/2023025/olm.

## References

[1] Alvey C. 1936. The morphology and development of the Monogenetic Trematode S*phyranura oligorchis* (Alvey 1933) and the description of *Sphyranura polyorchis* n.sp. Parasitology, 28, 229–253.

[2] Ayadi ZEM, Tazerouti F, Gastineau R, Justine JL. 2022. Redescription, complete mitochondrial genome and phylogenetic relationships of *Hexostoma thynni* (Delaroche, 1811) Rafinesque, 1815 (Monogenea, Hexostomatidae). Parasite, 29, 29. 35604345 10.1051/parasite/2022030 PMC9126124

[3] Bates J. 1997. The slide-sealing compound “Glyceel”. Journal of Nematology, 29, 565. 19274194 PMC2619815

[4] Bentz S, Combes C, Euzet L, Riutord JJ, Verneau O. 2003. Evolution of monogenean parasites across vertebrate hosts illuminated by the phylogenetic position of *Euzetrema* Combes, 1965 within the Monopisthocotylea. Biological Journal of the Linnean Society, 80, 727–734.

[5] Bentz S, Sinnappah-Kang ND, Lim LHS, Lebedev B, Combes C, Verneau O. 2006. Historical biogeography of amphibian parasites, genus *Polystoma* (Monogenea: Polystomatidae). Journal of Biogeography, 33, 742–749.

[6] Bernt M, Donath A, Jühling F, Externbrink F, Florentz C, Fritzsch G, Stadler PF. 2017. MITOS2 WebServer. 10.1016/j.ympev.2012.08.023 22982435

[7] Bernt M, Donath A, Jühling F, Externbrink F, Florentz C, Fritzsch G, Pütz J, Middendorf M, Stadler PF. 2013. MITOS: improved *de novo* metazoan mitochondrial genome annotation. Molecular Phylogenetics and Evolution, 69, 313–319. 22982435 10.1016/j.ympev.2012.08.023

[8] Berthier P, Du Preez LH, Raharivololoniana L, Vences M, Verneau O. 2014. Two new species of polystomes (Monogenea: Polystomatidae) from the anuran host *Guibemantis liber*. Parasitology International, 63, 108–119. 24090686 10.1016/j.parint.2013.09.014

[9] Bolger AM, Lohse M, Usadel B. 2014. Trimmomatic: a flexible trimmer for Illumina sequence data. Bioinformatics, 30, 2114–2120. 24695404 10.1093/bioinformatics/btu170 PMC4103590

[10] Bonett RM, Chippindale PT. 2006. Streambed microstructure predicts evolution of development and life history mode in the plethodontid salamander *Eurycea tynerensis*. BMC Biology, 4, 1–12. 16512919 10.1186/1741-7007-4-6 PMC1413558

[11] Bushnell B. 2017. BBMap Short Read Aligner. http://sourceforge.net/projects/bbmap.

[12] Caña-Bozada V, Llera-Herrera R, Fajer-Ávila EJ, Morales-Serna FN. 2021. Mitochondrial genome of *Scutogyrus longicornis* (Monogenea: Dactylogyridea), a parasite of Nile tilapia *Oreochromis niloticus*. Parasitology International, 81, 102281. 33401015 10.1016/j.parint.2020.102281

[13] Capella-Gutiérrez S, Silla-Martínez JM, Gabaldón T. 2009. trimAl: a tool for automated alignment trimming in large-scale phylogenetic analyses. Bioinformatics, 25, 1972–1973. 19505945 10.1093/bioinformatics/btp348 PMC2712344

[14] Chaabane A, Du Preez LH, Johnston GR, Verneau O. 2022. Revision of the systematics of the Polystomoidinae (Platyhelminthes, Monogenea, Polystomatidae) with redefinition of *Polystomoides* Ward, 1917 and *Uteropolystomoides* Tinsley, 2017. Parasite, 29, 56. 36562437 10.1051/parasite/2022056 PMC9879127

[15] Chaabane A, Verneau O, Du Preez LH. 2019. *Indopolystoma* n. gen. (Monogenea, Polystomatidae) with the description of three new species and reassignment of eight known *Polystoma* species from Asian frogs (Anura, Rhacophoridae). Parasite, 26, 67. 31746733 10.1051/parasite/2019067 PMC6865761

[16] Du Preez LH, Badets M, Verneau O. 2014. Assessment of platyhelminth diversity within amphibians of French Guiana revealed a new species of *Nanopolystoma* (Monogenea: Polystomatidae) in the caecilian *Typhlonectes compressicauda*. Folia Parasitologica, 61, 537–542. 25651695

[17] Du Preez LH, Domingues MV, Verneau O. 2022. Classification of pleurodire polystomes (Platyhelminthes, Monogenea, Polystomatidae) revisited with the description of two new genera from the Australian and Neotropical Realms. International Journal for Parasitology: Parasites and Wildlife, 19, 180–186. 36188110 10.1016/j.ijppaw.2022.09.004 PMC9519787

[18] Du Preez LH, Verneau O. 2020. Eye to eye: classification of conjunctival sac polystomes (Monogenea: Polystomatidae) revisited with the description of three new genera *Apaloneotrema* n. g., *Aussietrema* n. g. and *Fornixtrema* n. g. Parasitology Research, 119, 4017–4031. 33043418 10.1007/s00436-020-06888-w

[19] Emel SL, Bonett RM. 2011. Considering alternative life history modes and genetic divergence in conservation: a case study of the Oklahoma salamander. Conservation Genetics, 12, 1243–1259.

[20] Fan L, Xu W, Jia T, Netherlands EC, Du Preez LH. 2020. *Polystoma luohetong* n. sp. (Monogenea: Polystomatidae) from *Rana chaochiaoensis* Liu (Amphibia: Ranidae) in China. Systematic Parasitology, 97, 639–647. 32990886 10.1007/s11230-020-09937-1

[21] Hahn C, Bachmann L, Chevreux B. 2013. Reconstructing mitochondrial genomes directly from genomic next-generation sequencing reads – a baiting and iterative mapping approach. Nucleic Acids Research, 41, e129. 23661685 10.1093/nar/gkt371 PMC3711436

[22] Héritier L, Badets M, Du Preez LH, Aisien MSO, Lixian F, Combes C, Verneau O. 2015. Evolutionary processes involved in the diversification of chelonian and mammal polystomatid parasites (Platyhelminthes, Monogenea, Polystomatidae) revealed by palaeoecology of their hosts. Molecular Phylogenetics and Evolution, 92, 1–10. 26072314 10.1016/j.ympev.2015.05.026

[23] Hughes CR, Moore GA. 1943. *Sphyranura euryceae*, a new polystomatid monogenean fluke from *Eurycea tynerensis*. Transactions of the American Microscopical Society, 62, 286–292.

[24] Jin JJ, Bin Yu W, Yang JB, Song Y, De Pamphilis CW, Yi TS, Li DZ. 2018. GetOrganelle: a fast and versatile toolkit for accurate de novo assembly of organelle genomes. Genome Biology, 21, 1–31. 10.1186/s13059-020-02154-5 PMC7488116 32912315

[25] Kamegai S. 1971. On some parasites of a coelacanth (*Latimeria chalumnae*): a new Monogenea, *Dactylodiscus latimeris* ng, n. sp.(Dactylodiscidae n. fam.) and two larval helminths. Research Bulletin of the Meguro Parasitological Museum, 5, 1–5.

[26] Katoh K, Misawa K, Kuma KI, Miyata T. 2002. MAFFT: a novel method for rapid multiple sequence alignment based on fast Fourier transform. Nucleic Acids Research, 30, 3059–3066. 12136088 10.1093/nar/gkf436 PMC135756

[27] Kmentová N, Hahn C, Koblmüller S, Zimmermann H, Vorel J, Artois T, Gelnar M, Vanhove MPM. 2021. Contrasting host-parasite population structure: morphology and mitogenomics of a parasitic flatworm on pelagic deepwater cichlid fishes from Lake Tanganyika. Biology, 10, 797. 34440029 10.3390/biology10080797 PMC8389663

[28] Kritsky DC, Hoberg EP, Aubry KB. 1993. *Lagarocotyle salamandrae* n. gen., n. sp. (Monogenoidea, Polyonchoinea, Lagarocotylidea n. ord.) from the cloaca of *Rhyacotriton cascadae* Good and Wake (Caudata, Rhyacotritonidae) in Washington state. Journal of Parasitology, 79, 322–330.

[29] Landman W, Verneau O, Raharivololoniaina L, Du Preez LH. 2021. First record of *Metapolystoma* (Monogenea: Polystomatidae) from *Boophis* tree frogs in Madagascar, with the description of five new species. International Journal for Parasitology: Parasites and Wildlife, 14, 161–178. 33898217 10.1016/j.ijppaw.2021.01.012 PMC8056147

[30] Lanfear R, Frandsen PB, Wright AM, Senfeld T, Calcott B. 2017. Partitionfinder 2: new methods for selecting partitioned models of evolution for molecular and morphological phylogenetic analyses. Molecular Biology and Evolution, 34, 772–773. 28013191 10.1093/molbev/msw260

[31] Larsson A. 2014. AliView: a fast and lightweight alignment viewer and editor for large datasets. Bioinformatics, 30, 3276–3278. 25095880 10.1093/bioinformatics/btu531 PMC4221126

[32] Laslett D, Canbäck B. 2008. ARWEN: a program to detect tRNA genes in metazoan mitochondrial nucleotide sequences. Bioinformatics, 24, 172–175. 18033792 10.1093/bioinformatics/btm573

[33] Le TH, Blair D, Agatsuma T, Humair PF, Campbell NJH, Iwagami M, Littlewood DTJ, Peacock B, Johnston DA, Bartley J, Rollinson D, Herniou EA, Zarlenga DS, McManus DP. 2000. Phylogenies inferred from mitochondrial gene orders – a cautionary tale from the parasitic flatworms. Molecular Biology and Evolution, 17, 1123–1125. 10889225 10.1093/oxfordjournals.molbev.a026393

[34] Letunic I, Bork P, Gmbh BS. 2021. Interactive Tree Of Life (iTOL) v5: an online tool for phylogenetic tree display and annotation. Nucleic Acids Research, 49, 293–296. 10.1093/nar/gkab301 PMC8265157 33885785

[35] Mañé-Garzón F, Orlando G. 1962. Trematodos de las tortugas del Uruguay, V. Comunicaciones Zoológicas del Museo de Historia Natural de Montevideo, 94, 1–6.

[36] McAllister CT, Trauth SE, Hinck LW. 1991. *Sphyranura euryceae* (Monogenea) on *Eurycea* spp. (Amphibia: Caudata), from Northcentral Arkansas. Journal of the Helminthological Society of Washington, 58, 137–140.

[37] McAllister CT, Bursey CR, Steffen MA, Martin SE, Trujano-Alvarez AL, Bonett RM. 2011. *Sphyranura euryceae* (Monogenoidea: Polystomatoinea: Sphyranuridae) from the grotto salamander, *Eurycea spelaea* and Oklahoma salamander, *Eurycea tynerensis* (Caudata: Plethodontidae), in Northeastern Oklahoma, U.S.A. Comparative Parasitology, 78, 188–192.

[38] Monticelli FS. 1903. Per una nouva classificatione delgi “Heterocotylea”. Monitore Zoologico Italiano, 14, 334–337.

[39] Nguyen L, Schmidt HA, Von Haeseler A, Minh BQ. 2014. IQ-TREE: a fast and effective stochastic algorithm for estimating maximum-likelihood phylogenies. Molecular Biology and Evolution, 32, 268–274. 25371430 10.1093/molbev/msu300 PMC4271533

[40] Poche F. 1925. Das System der Platodaria. Archiv Für Naturgeschichte, 91, 1–459.

[41] Price E. 1939. North American monogenetic trematodes. IV. The family Polystomatidae (Polystomatoidea). Proceedings of the Helminthological Society of Washington, 6, 80–92.

[42] Price EW. 1938. North American monogenetic trematodes. II. The families Monocotylidae, Microbothriidae, Acanthocotylidae and Udonellidae (Capsaloidea). Journal of the Washington Academy of Sciences, 28, 183–198.

[43] Řehulková E, Mendlová M, Šimková A. 2013. Two new species of *Cichlidogyrus* (Monogenea: Dactylogyridae) parasitizing the gills of African cichlid fishes (Perciformes) from Senegal: morphometric and molecular characterization. Parasitology Research, 112, 1399–1410. 23403992 10.1007/s00436-013-3291-9

[44] Seemann T, Booth T. 2018. Barrnap: BAsic Rapid Ribosomal RNA Predictor. https://github.com/tseemann/barrnap.

[45] Shen W, Le S, Li Y, Hu F. 2016. SeqKit: a cross-platform and ultrafast toolkit for FASTA/Q file manipulation. PLoS One, 11, 1–10. 10.1371/journal.pone.0163962 PMC5051824 27706213

[46] Sinnappah ND, Lim LHS, Rohde K, Tinsley R, Combes C, Verneau O. 2001. A paedomorphic parasite associated with a neotenic amphibian host: phylogenetic evidence suggests a revised systematic position for Sphyranuridae within anuran and turtle Polystomatoineans. Molecular Phylogenetics and Evolution, 18, 189–201. 11161755 10.1006/mpev.2000.0877

[47] Sitko J, Koubkova B. 1999. A simple differentiation of two genera *Brachylecithum* and *Lutztrema* (Trematoda: Dicrocoeliidae) based on Borax carmine and Astra blue staining method. Helminthologia, 36, 119–121.

[48] Solà E, Álvarez-Presas M, Frías-López C, Littlewood DTJ, Rozas J, Riutort M. 2015. Evolutionary analysis of mitogenomes from parasitic and free-living flatworms. PLoS One, 10, 1–20. 10.1371/journal.pone.0120081 PMC4368550 25793530

[49] Tinsley RC, Tinsley MC. 2016. Tracing ancient evolutionary divergence in parasites. Parasitology, 143, 1902–1916. 27576454 10.1017/S0031182016001347

[50] Di Tommaso P, Moretti S, Xenarios I, Orobitg M, Montanyola A, Chang JM, Taly JF, Notredame C. 2011. T-Coffee: a web server for the multiple sequence alignment of protein and RNA sequences using structural information and homology extension. Nucleic Acids Research, 39, 13–17. 10.1093/nar/gkr245 PMC3125728 21558174

[51] Tumlison R, McAllister CT, Robison HW, Connior MB, Sasse DB, Cloutman DG, Durden LA, Bursey CR, Fayton TJ, Schratz S, Buckley M. 2017. Vertebrate natural history notes from Arkansas. Journal of the Arkansas Academy of Science, 71, 7–16.

[52] Vassilieva AB, Poyarkov NA, Iizuka K. 2013. Pecularities of bony skeleton development in Asian clawed salamanders (*Onychodactylus*, Hynobiidae) related to embryonization. Biology Bulletin, 40, 589–599.

[53] Verneau O, Bentz S, Sinnappah ND, Du Preez LH, Whittington I, Combes C. 2002. A view of early vertebrate evolution inferred from the phylogeny of polystome parasites (Monogenea: Polystomatidae). Proceedings of the Royal Society B: Biological Sciences, 269, 535–543. 10.1098/rspb.2001.1899 PMC1690918 11886648

[54] Verneau O, Du Preez LH, Badets M. 2009. Lessons from parasitic flatworms about evolution and historical biogeography of their vertebrate hosts. Comptes Rendus – Biologies, 332, 149–158. 19281948 10.1016/j.crvi.2008.08.019

[55] Williams J. 1995. Phylogeny of the Polystomatidae (Platyhelminthes, Monogenea), with particular reference to *Polystoma integerrimum*. International Journal for Parasitology, 25, 437–441. 7635619 10.1016/0020-7519(94)00138-e

[56] Wilm A, Higgins DG, Notredame C. 2008. R-Coffee: a method for multiple alignment of non-coding RNA. Nucleic Acids Research, 36, e52. 18420654 10.1093/nar/gkn174 PMC2396437

[57] Zhang J, Wu X, Li Y, Zhao M, Xie M, Li A. 2014. The complete mitochondrial genome of *Neobenedenia melleni* (Platyhelminthes: Monogenea): mitochondrial gene content, arrangement and composition compared with two *Benedenia* species. Molecular Biology Reports, 41, 6583–6589. 25024046 10.1007/s11033-014-3542-6

